# Patients’ preferences for delivering bad news in palliative care in Ethiopia: a qualitative study

**DOI:** 10.1186/s12904-023-01275-5

**Published:** 2023-11-03

**Authors:** Ephrem Abathun Ayalew, Ditaba David Mphuthi, Kholofelo Lorraine Matlhaba

**Affiliations:** Department of Health Studies, University of South Africa, Pretoria, South Africa

**Keywords:** Breaking bad news, Ethiopia, Life-threatening illness, Palliative care, Preferences

## Abstract

**Background:**

One of the major challenges for healthcare professionals relates to awareness of patients’ preferences relative to how and when to break bad news and how much information should be disclosed in the eventuality of a serious medical diagnosis or prognosis. On occasions, a serious medical diagnosis or prognosis is withheld from the patient. There is a scarcity of evidence about cultural preferences regarding breaking bad news in the palliative care setting in Ethiopia. Therefore, it is necessary to understand the surrounding cultural issues to properly convey bad news. The purpose of the study was to explore Ethiopian patients’ cultural preferences for receiving bad news in a palliative care setting.

**Methods:**

A qualitative research approach and nonprobability, purposive sampling method were applied. In-depth interviews were employed to collect data from eight patients who were diagnosed with cancer and cancer with HIV/AIDS during the time of data collection. Thematic analysis was applied to identify themes and subthemes. The data were transcribed verbatim and analysed using ATLAS.ti 22 computer software.

**Results:**

The following three themes emerged and are reported in this study: (1) Perceptions about life-threatening illness: religious values and rituals are essential for establishing perspectives on life-threatening illnesses and preferences in receiving bad news. (2) Experiences with life-threatening illness: study participants’ experience with the method of breaking bad news was sad, and they were not provided with sufficient details about their medical condition. Making appropriate decisions, fulfilling the ordinance of religious faith, and avoiding unnecessary costs were outlined as benefits of receiving bad news. (3) Preferred ways of breaking bad news; the findings revealed that incremental, amiable and empathic methods for delivering bad news were preferred. It was suggested that the presence of family members is crucial when receiving bad news.

**Conclusion:**

Patients choose to be told about their medical conditions in the presence of their family. However, the patient’s needs for receiving bad news were unmet. Patients should be involved in the treatment decision process. Delivery of bad news needs to tailor the preferred methods, cultural values, and religious beliefs. Delivering bad news according to the patients’ preferences helps to fulfil their wishes in palliative care.

**Supplementary Information:**

The online version contains supplementary material available at 10.1186/s12904-023-01275-5.

## Background

Noncommunicable diseases (NCDs) are the main causes of higher death rates in lower income countries [1]. According to the World Health Organization (WHO), the number of people in need of palliative care each year is estimated to be 40 million, with 78% of the people living in low- and middle-income countries. Nevertheless, only 14% receive the service globally [2]. In line with this, healthcare professionals are often faced with breaking bad news to the patient and/or family.

Ethiopia is “a country in east-Africa” [3]. The prevalence of NCDs has become a public health issue in Ethiopia, resulting in a high death and morbidity rate, and NCDs have become an additional burden on infectious diseases. NCDs account for 39% of all deaths, and 275 000 premature deaths occurred in 2016 [4, 5]. According to GLOBOCAN, the number of deaths due to cancer was 51 865 in 2020 in Ethiopia [6]. With the rise of NCDs, the demand for palliative care is increasing, and late-stage presentations and delays in the diagnosis of life-threatening illnesses such as cancer are common [7]. According to research conducted in Ethiopia, 73% of cancer patients present to the hospital with late-stage disease, which results in a high need for palliative care; the majority of patients receive palliative radiotherapy, and patients continue to suffer from pain and symptoms, as the waiting time for radiotherapy is long [8].

Palliative care, as defined by the WHO, is care for patients with life-threatening illness that aims to improve the quality of life of patients by preventing and alleviating suffering, not only physical pain but also psychological, social and spiritual problems. Furthermore, palliative care helps patients to live as actively as possible while accept death as a normal process [2]. Life-threatening illness refers to an illness “capable of causing death or has a serious effect on the life of the patient” [9]. In Ethiopia, there are various milestones taken to make palliative care available in public hospitals. Short course trainings on palliative care were given for healthcare professionals, and national palliative care manuals were prepared and endorsed. Some hospitals integrate into their hospital and provide palliative care services. However, the service is insufficient and not yet accessible for many patients suffering from life-threatening illness, and the Ethiopian medical education system does not currently include palliative care. The burden of moderate to severe pain predominates, significantly influencing the lives of patients and their families [10, 11]. Pain is frequently overlooked and undertreated despite the prevalence being high [12, 13]. Morphine is included in the World Health Organization (WHO) model list of essential medications for the use of pain and palliative care [14]; however, oral morphine is poorly available and accessible. The patients’ needs for care, psychosocial support and symptom management are not satisfied [12]. Furthermore, a study in Ethiopia showed that the healthcare professionals’ method of breaking bad news does not meet the patients’ preferences [15].

The Ethiopian proclamation declares that “health professionals” should inform patients about their serious medical condition [16]. Doctors are responsible for breaking bad news when life-threatening illness is diagnosed in Ethiopia. However, nurses who received specific training could also break bad news in some cases and situations, example a diagnosis of HIV/AIDS. According to a study conducted in Ethiopia, healthcare workers have good knowledge and favourable attitudes towards end-of-life care and palliative care, but the practice remains poor [17]. Evidence also showed that the majority of physician participants are not aware of the SPIKES protocol for breaking bad news and have not received training on breaking bad news [15].

Patients with life-threatening illness might benefit much from palliative care, but it cannot be achieved without properly breaking bad news, which requires understanding cultural preferences. Breaking bad news refers to conveying or communicating bad news to a patient or next of kin [18]. Studies in non-African countries reveal that patients prefer to be informed and want to have as much information as possible concerning their diagnosis, present health status and treatments [19, 20]. Similarly, a study conducted in Ethiopia revealed that patients want to be informed of their diagnosis. Nevertheless, patients’ preferences for the disclosure of bad news vary from those of family caregivers, who want that only family members be told [21]. In African countries, the next of kin have a primary role to be informed and are often the decision makers when a life-threatening illness is diagnosed [22]. There are recommended guidelines for breaking bad news in the literature. The SPIKES protocol recommends setting the physical and other situations, assessment for perception, asking for wanting or not and on the amount of information, informing and summarizing the conversation [23]. The following six steps are advised by a different model: assessment, planning, preparation, disclosure, support, and conclusion [18].

In the Ethiopian context, the next of kin often withhold bad news from the patient to protect their loved ones from news on terminal illnesses. As a result, serious medical information is frequently withheld from the patient when a life-threatening illness is diagnosed [7, 22]. This affects the fulfilment of patients’ needs and the relationship between patients and families, as collusion leads to the setting of different goals. Furthermore, it influences patient involvement in medical decisions.

Present knowledge about patients’ preferences regarding how they would like to be told, who should be told and how much detail they want to know relating to their condition is poor in Ethiopia. The major challenges for healthcare professionals are patient preferences in regard to how and when to break bad news, how much information should be given, and to whom bad news should be delivered when a serious medical diagnosis or prognosis is made. In this study, preferences for breaking bad news pertain to choices on how bad news should be disclosed. There is a paucity of evidence about preferences regarding disclosing bad news in palliative care settings in Ethiopia. Therefore, the knowledge gap regarding whether the patient prefers bad news to be disclosed to the family or directly to the patient or together with the family and how to deliver bad news properly needs to be uncovered.

The purpose of the study was to explore Ethiopian patients’ cultural preferences for receiving bad news in a palliative care setting. The research questions were: What are the preferences of individuals regarding breaking bad news in palliative care when diagnosed with life-threatening illness? What are the needs for information related to breaking bad news when patients are diagnosed with life-threatening illness?

## Methods

### Design and setting of the study

A qualitative, ethnographic research approach was used to explore the views, experiences, preferences and cultural issues of the study participants about breaking bad news. This approach is chosen because the study seeks to generate descriptive data from people with their own views, and it allows for an in-depth understanding of breaking bad news. Qualitative research enabled us to explore and describe the phenomenon in its context in depth. The study was performed at St Paul’s Hospital and Hospice Ethiopia in Addis Ababa, Ethiopia. St. Paul’s Hospital is one of the largest referral hospitals under St. Paul’s Hospital Millennium Medical College. It has an inpatient facility with 700 beds and sees an average of 1200 emergency and outpatient patients per day [24]. Hospice Ethiopia is an indigenous nongovernmental organization working in the area of comprehensive palliative care for people diagnosed with life-threatening illness in Addis Ababa. The organization provides its services in home-based care and ambulatory care settings with two nurses, one clinical/health officer trained in palliative care and 9 administrative staff members. These study settings are selected purposefully because they work in the area related to this research phenomenon and entail expertise in the study phenomenon, experienced participants and accessibility.

### The study participants and sampling methods

The study participants were patients with life-threatening illness. They were selected by using a nonprobability purposive sampling method. A purposive sampling approach is used in this research because it helps select key informants who have knowledge about the study topic and can provide detailed information. The sample size was eight, which was determined by data saturation. The number of study participants was determined when there was no new information during the data collection process. When analysing qualitative data, 84% of data can be coded at the interview number of 6 and 89% at the interview number of 6, and the number of new codes is small after the sixth sample [25]. Data collection and analysis processes were performed concurrently, and when the same information was revealed during interviews and analysis, the researcher stopped recruiting participants for this study.

### Inclusion criteria

Patients diagnosed with life-threatening illness who had full insight into their condition and no cognitive impairment were included. Mentally incompetent patients who were not informed of their medical conditions, minors who were less than 18 years old and people who were seriously ill were not included in the study. Cancer patients with or without HII/AIDS who had insight into their condition were accessible and involved in the study.

### Data collection methods

Data were collected through in-depth interviews by using semi structured interview guide questions that were developed for this study based on the study purpose and objectives. The interviews were conducted at the patient’s home and the study areas depending on the choice of the participants. The principal researcher collected the data between December 2021 and July 2022. All interviews were recorded by using an audio recorder following written consent. In-depth interviews were chosen for this research because they are appropriate for exploring personal experiences, perceptions, and beliefs and obtaining in-depth information. The rigor of this research was maintained by member checks, peer debriefing and methodological alignment of the research.

### Data analysis

A thematic data analysis was applied to describe the results. Both manual and computer-assisted data analysis approaches were carried out, and hence, the ATLAS.ti 22 data analysis software system was used. This computer program assisted in sorting, coding and categorizing the data. The analysis process entailed the preparation and organization of data, coding, categorization and theme development. The computer program was used for the majority of the analysis work. In addition, after the data were coded and categorized, the working paper was printed, and the associated ideas were reread and categorized manually using colors. Data analysis was performed by the principal researcher, and the process included listening to the audio record and rereading the transcribed English version for ultimate familiarization with the data and establishing patterns.

For this research, the data analysis included the following procedure: Preparation and organization of data: the data were transcribed, grouped, and assigned to a category. During preparation and organizing, the researcher was better familiarized with the data and immersed in the data by reading and rereading the transcribed data to find similarities and differences. Coding: The data were coded based on categories. This was done as soon as data collection and analysis began. Categorizing and developing themes: Many categories were developed at this step. The researcher was immersed with the data, reread and recoded to reach meaningful themes and subthemes for the ultimate descriptive results.

## Results

The patients’ medical diagnoses included cancer of the breast, colon, oesophagus, prostate, cervix, liver, and HIV/AIDS. Their religious background was Orthodox Christian, Evangelical Christian (Protestant) and Muslim. The study participants’ residencies were from both rural and urban areas. The patient participants’ educational levels ranged from no formal education to diploma holders, and their ages ranged from 36 to 70 years old. Participants with no formal education were helped to understand the questions by describing the purpose of the study at their pace and before the interview. Moreover, adequate time for questions and answers was given, and the interview was conducted in the local language, Amharic. Table 1 shows the demographic information of the study participants:

**Table 1:** Study participant demographic information

Characteristics		Number of participants (N = 8)
Sex	Female	5
	Male	3
Age	35–45	3
	46–55	2
	56–65	2
	70–80	1
Diagnosis	Cancer	6
	Cancer and HIV/AIDS	2
Marital status	Married	4
	Widowed	1
	Divorced	3
Religion	Protestant Christian	2
	Muslim	1
	Orthdox Christian	5
Education	Diploma	2
	High school graduate	3
	Less than high school	3
Residence/location	Addis Ababa	5
	Outside of Addis Ababa	3

In this section, three themes emerged from interviews and are discussed with the support of direct quotations from the patients. Table 2 depicts the themes and subthemes that emerged from the study as follows.

**Table 2:** Themes and subthemes for patients

Themes	Subthemes
Theme 1. Perceptions about life-threatening illness	1.1. Social values, beliefs and attitudes regarding life-threatening illness 1.2. Religion, faith, and religious rituals play an important role in conveying bad news
Theme 2. Experiences with life-threatening illness	2.1. Responses to bad news disclosure 2.2. Unmet needs of information on the patients’ serious medical conditions 2.3. Benefits of breaking bad news 2.4. Coping mechanisms with bad news
Theme 3. Preferred ways of breaking bad-news	3.1. Individual preferences when breaking bad news 3.2. Culturally acceptable manners of breaking bad news 3.3. Inappropriate and unacceptable ways of breaking bad news

### Theme 1. Perceptions about life-threatening illness

Religious faith, beliefs and rituals are invaluable cultural attributes when serious news is discussed. Participants in the study reported that their views of health and illness were framed by their religious faith and beliefs.

### Social values, beliefs and attitudes regarding life-threatening illnesses

Suffering is influenced by one’s perceptions of health and illness. Most participants in this study perceived illness from their religious belief point of view:

*“Serious illness belongs to Satan; it is not ours. Because it belongs to demons, it may be taken off from the patient in God’s day. At the time that God permits, it will be taken away. I will be saved. I may get sick, but I will be saved in God. The doctor’s role is to diagnose the disease.” (I1P1PT).*

*“…this disease does not mean anything. There is no need to depart from the Creator and to be discouraged by the serious illness. Satan is the one who breaks the spirit… God might allow the illness to strengthen our faith through test…. God has given me a serious illness, which may be because my sins are too great; however, I can find a solution from Him by requesting His mercy” (I3P3PT).*

### Religion, faith, and religious rituals play an important role in conveying bad news

Patients have described that their faith in God and religious rituals are fundamental to stay encouraged, to obtain meaning in life and to maintain hope.

*“The doctor told me that the cancer was stage four and that my chances of survival were very low. When he told me this, I did not accept and believe him because life is in God’s hands. I will live as long as God allows me. I am taking the medicines properly and pray passionately in my religion. Life belongs to God…” (I4P4PT).*

*“I was desperate and stopped taking my medicines; it was through God’s encouragement during prayers that I restarted taking the drugs. Nothing will happen in my capacity when I live in this world… You need to have God more than humans. You should praise God; when you thank God, then the blessing of health will be added to you… Whether I am cured from my illness or not, I thank God very much…” (I1P1PT).*

### Theme 2. Experiences with life-threatening illness

This theme describes experiences regarding receiving bad news, how to overcome problems related to life-threatening illness, unfulfilled needs of patients, and the benefits of knowing the health status based on the patients’ experience in the following subthemes:

### Responses to bad news disclosure

The participants reported that they had various emotional responses against breaking bad news.

*“I was broken down and in tears when I received the bad news, and I was in panic as I walked to tell my family. The information I had and my perception of cancer were both frightening. When I was informed, I was shocked. I cried, I had not eaten properly, and I withdrew from social life for several weeks. I was furious and vented my rage on my close family and friends. When the doctor informed me of the diagnosis, I said okay and ran away” (I2P2PT).*

*“… When I found myself in the position of having cancer, I felt sad and uncomfortable. I was angry and insulted people. I was terrified and concerned about the disease. My life has been full of frustration; I have lost my attention to social relationships, and I did not get along with people; I have become an easily upsetting person, and I have quarrelled with family and friends for no reason. I also stopped working at that time” (I1P1PT).*

### Unmet needs of information on the patients’ serious medical conditions

The study participants reported that their needs for information and support in breaking bad news were not met:

*“I asked them to explain it to me as I did not have enough information…They did not provide me with enough information. Every time I saw my treating doctor, he provided me with insufficient information and talked to me briefly. They rushed up to see the next patient” (I10P10PT). “…The doctor told me the cancer was in stage four and I had no idea what that meant. He never described it. The way he delivered the bad news hurt me more than the disease itself. Patients I met at the hospital provided me with important information about the side effects of chemotherapy. They also encouraged me and gave me advice on how to proceed with the treatments” (I10P10PT).*

### Benefits of breaking bad news

This study showed that breaking bad news benefits the patient in many ways, such as making informed choices to conduct religious sacred rites and minimizing or avoiding unnecessary costs.

*“If the patient is informed of the illness, she/he can decide what to do by herself. If the disease is at a higher stage for cure, she/he will decide on the treatment. It is beneficial to be aware of the outcome of the disease and interventions so that you can make informed decisions” (I3P3PT).*

*“If you know your medical fact, you would beg the Creator for mercy, and you may pray for healing. I will be more committed and look for solutions in my faith if I am aware of the bad news” (I3P3PT).*

#### Coping mechanisms with bad news

Patients have described that religious beliefs enable them to accept bad news and overcome challenges associated with a life-threatening illness. Family, friends and healthcare professional support and hospice care were also useful.

*“It is not by my capacity that I overcome my challenges. To be honest, it is God who has helped me in any way. It is God who has encouraged me; it could not happen in my capacity. God gave me the strength” (I1P1PT).*

*“I think, my neighbours care, and support assisted me in surviving. Everyone reassured me; the social support makes me stronger and encouraged. Truly speaking the doctor’s become my hope and truly supported me. I think God has put them (the healthcare professionals) in a good position for me. They were very good. They care for people very much” (I2P2PT).*

### Preferred ways of breaking bad news

This theme explains the preferences of patients regarding breaking bad news and their chosen methods for breaking bad news.

### Individual preferences when breaking bad news

Patients prefer to be informed of their illness in detail, and they want the presence of their family when receiving bad news:

*“I would like to know my medical condition in depth. They have done laboratory tests for me, and I took chemotherapy and have follow-ups, but I do not know the stage of my illness. I should have been informed, but I was not… Bad news should be told for the patient” (I3P3PT).*

*“Bad news should be conveyed with the family. Family should be involved during bad news conversations. The doctor also needs to involve family when planning treatments and treating so that patient can get complete support” (I1P1PT).*

### Culturally acceptable ways of breaking bad news

The preferred approaches for breaking bad news were gradual, amiable, and delivering in a compassionate manner. Empathetic approach and not intensifying when presenting bad news were also emphasized.

*“I would like (the news) to be told the bad news like a family manner with the healthcare providers; friendly approach, with love, not much formal. The health professional needs to be prepared how to approach and explain to the patient like a brother or a sister, provide counselling support and describe the issue with example” (I4P4PT).*

### Gradual manner

*“Bad news should be told in roundabout manner and presentation should be light; take a long process by talking about general things before coming to the point. When explaining, I want to sit down and receive little information. My doctor informed me abruptly that it was cancer and I have to do surgery and take chemotherapy… It was hard to hear that way. I wish the doctor told me it was a tumour and explained how it turned into serious illness or cancer”* (I5P5PT).

### Empathetic approach

*“When you tell bad news, reassure and tell him in a gentle way. Any patient is happy to find a doctor who gives reassurance. I visited different hospitals to see doctors due to my illness. I would consider a good doctor if they effectively communicated and gave love”* (I3P3PT).

*“The doctor told me that the cancer was at a good stage and I will be cured by faith. He advised me to keep my hope, but I have had cough as my lung was affected. ……they gave me medicines” (I2P2PT).*

### Inappropriate and unacceptable ways of breaking bad news

Telling prognostication was not accepted by the majority of patient participants, while poor prognosis was preferred to be told to the family member. Moreover, patients do not accept a direct disclosure of serious news, i.e., telling without preparation, adequate information about the illness, reassurance and empathy:

*“While it is good to inform bad news (diagnosis), telling the estimated time of death is not acceptable, as life is in God’s hands. I strongly oppose predicting the time of death. The doctor said to my family, “she will not survive, she has only one month to live”, and I was sent for Hospice care; however, I lived logger” (I4P4PT).*

*“When I was informed of my diagnosis, the doctor’s manner of communication shocked me. I think the healthcare professionals speak abruptly without preparing the patient, and it feels as if something terrible thing happened instantly. I never thought it was cancer. I thought it was a tumor. I took it something light, but when he told me it was cancer, I was so shocked” (I5P5PT).*

### Experiences breaking bad news

The patient’s experience in receiving bad news was sad, and the utterances were upsetting:

*“The doctor’s discourse was bad, the way he approached and terms he used to inform me were disappointing. He informed me of the problem directly… The approach for telling bad news was disgusting. It is immoral to deliver bad news without a proper way” (I1P1PT).*

*“…My doctor said, the swelling on the tip of your rectum is cancer without preparing and taking care. I and my family were tuned in and could not believe it. It was tough for me, and I felt desperate as I had not anticipated. I was upset by his method of telling and the news… When the doctor continued speaking to me, I could not listen to him…” (I10P10PT).*

## Discussion

Patients stated that their faith in God and religious rituals are fundamental for staying encouraged, seeing some meaning in life amidst suffering from incurable diseases, and holding onto the hope that things shall improve. Ethiopians have deep religious faith and, for the majority, believe in God [26]. This study confirmed that patients cope with their challenges and difficult times related to receiving bad news and their serious illness with their religious beliefs and rituals. As a result, it has been suggested that breaking bad news conversations and discourses must incorporate these aspects of cultural values. In line with this, studies have reported that patients want health professionals to regard and consider their religious values [19].

This study outlined the preferred methods for breaking bad news. Telling gradually; telling a small amount of information step by step, telling indirectly (going roundabout) to show caution. Furthermore, patients want to be psychologically and emotionally prepared and provided with warning statements before the disclosure of bad news, and they refused a direct approach for conveying bad news. Similarly, a quantitative study in Ethiopia showed that patients want to be consulted on how much information they should be told and be informed progressively [15]. However, some patients may prefer to be informed without delay to begin treatment as soon as possible [27].

The empathic approach was the other method suggested by the participants of this study. Hence, humility was voiced as a fundamental ethical practice, and patients should be supported to be calm and reassured emotionally when delivering bad news. The word “Ayzoh/sh”, encouraging and comforting words and affirmative words are greatly appreciated in the consolation of participants in this study. However, patients reported that healthcare professionals did not demonstrate adequate empathy [15]. The amiable approach is also a culturally suggested method of telling bad news. Patients want to build good relationships with their care provider prior to talking about bad news and prefer friendly approaches when bad news is delivered. Showing respect, empathetic approach and humbleness are culturally valued as therapeutic communication [28]. Using comforting words, culturally suitable phrases, and reassurance and encouraging religious rituals to maintain faith in their creator/god are central to maintaining hope and cultural values when delivering bad news.

In this study, most patients wanted to know their medical condition in detail. They wanted to obtain some information about the illness and treatment options and discuss the causes or factors that contributed to the disease. This helps them to address their uncertainties and involvement in medical decisions. Patients also preferred to receive bad news in the presence of their family. This finding is consistent with those of a study performed in Ethiopia, which found that patients desired to discuss their medical conditions in the presence of family members [15]. They have, however, reported receiving insufficient information about their medical condition, as well as a negative experience with receiving bad news. Similarly, a quantitative study showed that patients with life-threatening illness scored low levels of satisfaction with the information provided about their illness and breaking bad news approaches in Ethiopia [15].

It is culturally inappropriate to tell the patient about a poor prognosis directly and prefer to receive it in the presence of family; however, some patients want to represent their family. According to a study conducted in Ethiopia, patients prefer to delegate their family when dealing with their disease prognosis [21]. Illness and health are viewed from the spiritual and religious points of view [29]. Thus, it is believed that God has the power to determine how long a person lives. It is therefore culturally inappropriate to tell prognostications, and patients did not prefer to be told how long they have left to live in this study. Often, a family member is delegated to deal with serious medical conditions and, if needed, to organize a meeting with the patient [30].

The benefits of delivering bad news for the patient include fulfilling the religious ordinance. Prayers, using holy water and healing are the remedies patients seek when serious illness is diagnosed in Ethiopia [31, 32]. Patients present to healthcare facilities at a late stage of their illness and pay for treatments that are likely to be futile [7, 33]. Hence, knowing the conditions and objectives of serious treatments helps them to avoid costs for futile treatments and make the right decisions. Providing knowledge on treatment possibilities assists patients in making informed decisions [34]. Nevertheless, current practice is that serious medical news is concealed from patients [30]. Similarly, this study verified that patients did not receive adequate information on their medical condition. Breaking bad news also allows one to discuss emotional issues, psychological pain, distress, and fears and to obtain help. The findings from this study were consistent with those of other studies [35–38].

## Conclusion

Patients with life-threatening illnesses need to be informed of their serious medical conditions in detail. The majority of patients preferred to know their serious medical reports accompanied by their families and be involved in their medical decision process. Patients were dissatisfied with the present approaches to conveying bad news. Telling bad news directly and prognostication are not accepted. Incremental, amiable, and empathetic approaches for delivering bad news should be followed. Therefore, serious medical news should be delivered gradually rather than broken.

Patients want their religious values to be taken into account when receiving bad news. Culturally acceptable and preferred terms are desired by patients when receiving bad news. Appropriate delivery of bad news provides the advantages of helping patients become involved and make informed medical decisions, fulfil their needs and reduce distress in palliative care settings. Nevertheless, the existing practice of breaking bad news does not satisfy patients’ preferences and information needs. Therefore, delivering bad news guidelines should tailor the choices of patients, and culturally sensitive guidelines for breaking bad news should be available in the Ethiopian context.

### Electronic supplementary material

Below is the link to the electronic supplementary material.

Supplementary Material 1

## Data Availability

Data generated during the study and that were analysed and used to produce the study results are available for access. Ephrem Abathun can be contacted through abathunephrem@yahoo.com OR 10342435@mylife.unisa.ac.za should there be a request for the data from this study.
