# Supplementary material for: Patients’ preferences for delivering bad news in palliative care in Ethiopia: a qualitative study
Source: BMC Palliat Care. 2023 Nov 3;22:170. doi: 10.1186/s12904-023-01275-5 (PMC10623733; doi:10.1186/s12904-023-01275-5)
Supplement: Supplementary file 1 — Supplementary Material 1 [file 12904_2023_1275_MOESM1_ESM.docx]

**DATA COLLECTION TOOLS**

**Interview guide: Patients diagnosed with life-threatening illness**

Dear participant, my name is Ephrem Abathun and I am a clinical/health officer working with Hospice Ethiopia. I am the principal researcher for the research titled with DEVELOPMENT OF CULTURALLY SENSITIVE GUIDELINES FOR BREAKING BAD NEWS IN PALLIATIVE CARE: ETHIOPIA at the University of South Africa.

Thank you for taking the time to participate in this study. Let us discuss about your cultural views, needs of information and preferred ways of bad news communication.

1. Would you tell me about your biographic details please?
2. I understand that you have a diagnosis of life-threatening illness, would you tell me how you would like to be told the bad news?

*Prompt:*

How much details would you like to know about your medical condition?

Who do you think should be told the bad news?

Who would you think should make a decision on treatment?

Would you tell me your experience with your doctor or nurse in disclosure of your medical diagnosis/prognosis and what helps you to cope with your situation?

*Prompt:*

How would you think the cultural perspectives that your doctor or nurse should see during disclosure of bad news?

What would you prefer to be taken in to account when you receive the bad news?

Would you have any other opinion?
